# ﻿Metaphase chromosomes of five Neotropical species of the genus *Drosophila* (Diptera, Drosophilidae)

**DOI:** 10.3897/compcytogen.17.108265

**Published:** 2023-12-05

**Authors:** Doris Vela, Erika Villavicencio

**Affiliations:** 1 Pontificia Universidad Católica del Ecuador, Facultad de Ciencias Exactas y Naturales, Laboratorio de Genética Evolutiva. Av. 12 de Octubre 1076 y Roca, Quito, Ecuador Pontificia Universidad Católica del Ecuador Quito Ecuador

**Keywords:** Andean, *Drosophila* chromosomes, *guarani*, *mesophragmatica*, metaphase, *tripunctata*

## Abstract

The mitotic metaphases of five Andean species of genus *Drosophila* are described for the first time. The evolutionary and interspecific genetic relationships within three Neotropical *Drosophila* species groups are analyzed. The diploid chromosome number for each species is as follows: *D.cashapamba* Céspedes et Rafael, 2012 2n = 6 (2V, 1J) (X = J, Y = R), *D.ecuatoriana* Vela et Rafael, 2004 2n = 10 (3R, 2V) (X = V, Y = R), *D.ninarumi* Vela et Rafael, 2005 2n = 10 (3R, 1V, 1D) (X = V, Y = R), *D.urcu* Vela et Rafael, 2005 2n = 12 (4R, 2V) (X = V, Y = R), *D.valenteae* Llangarí-Arizo et Rafael, 2018 2n = 8 (3R, 1J) (X = J, Y = R).

## ﻿Introduction

The ancestral karyotype for the genus *Drosophila* Fallén, 1823 (Diptera, Drosophilidae) consists of five pairs of large chromosomes (V shape or J shape) and one pair of dots (Sturtevant and Novitski 1941). This *Drosophila* metaphase chromosome configuration has been commonly observed, for instance, in some species of Neotropical groups of the type subgenus Drosophila: *D.guarani* group (King 1947), *D.mesophragmatica* group (Brncic and Koref 1957; Hunter and Hunter 1964), *D.repleta* group (Wasserman 1960) and *D.tripunctata* group (Pipkin and Heed 1964). The species of the type subgenus present a chromosome configuration ranging from three to six pairs of chromosomes. Cytogenetics studies demonstrated that in the genus *Drosophila* karyotypes of species may differ from the ancestral karyotype by the number of chromosomes and the chromosomal configuration, but chromosomal rearrangements do not break the integrity of Muller elements (chromosome arms and associated linkage groups) (Schaeffer 2018).

By means of the karyotypes, it is possible to observe the chromosomal rearrangements (inversions, translocations, duplications etc.) in species, and how they can limit the genetic exchange and potentially drive speciation (Noor et al. 2001). In addition, it is possible to detect interspecific and intraspecific polymorphism in species of *Drosophila* (Deng et al. 2007). Therefore, karyotypes are an important tool for understanding the evolutionary history of the *Drosophila* species, to conduct comparative genomics studies and to allow genome assembly at the chromosome level (Schaeffer 2018).

Most of the available cytological data about Neotropical species of *Drosophila* were reported in the past century (Metz and Moses 1923; Patterson and Wheeler 1942; Wharton 1943; Burla et al. 1949; Clayton and Wasserman 1957; Clayton and Wheeler 1975). In the most recent cytological studies of Neotropical species of *Drosophila* karyotypes of ten species from four sibling species groups have been described: *D.chorlavi* Céspedes et Rafael, 2012, *D.mesophragmatica* Duda, 1927 and *D.rucux* Céspedes et Rafael, 2012 from the *D.mesophragmatica* group (Mafla 2012), *D.butantan* Ratcov, Vilela et Goñi, 2017, *D.sachapuyu* Peñafiel-Vinueza et Rafael, 2018, and *D.zamorana* Peñafiel-Vinueza et Rafael, 2018 from the *D.guarani* group (Ratcov et al. 2017; Vela and Villavicencio 2021), *D.huancavilcae* Rafael et Arcos, 1989, *D.inca* Dobzhansky et Pavan, 1943, and *D.yangana* Rafael et Vela,;2003 from the *D.repleta* group (Mafla 2005, 2008), and *D.montevidensis* Goñi et Vilela, 2016 from the *D.tripunctata* group (Goñi and Vilela 2016).

In this study, the karyotypes of five Andean species of *Drosophila* from three sibling species groups are described for the first time: *D.ecuatoriana* Vela et Rafael, 2004 and *D.valenteae* Llangarí-Arizo et Rafael, 2018 from the *D.guarani* group, *D.cashapamba* Céspedes et Rafael, 2012 from the *D.mesophragmatica* group, *D.ninarumi* Vela et Rafael, 2005 and *D.urcu* Vela et Rafael, 2005 from the *D.tripunctata* group.

## ﻿Methods

### ﻿Species stock

The species analysed correspond to natural populations of: *D.cashapamba* (QCAZ-I 2349), Sangolquí Canton (location 0°19'59.3"S, 78°25'51"W DMS); *D.ecuatoriana* (QCAZ-I 1609), Yanacocha Forest (location 0°7'3.8"S, 78°35'9.4"W DMS); *D.ninarumi* (QCAZ-I 1765), Cruz Loma Forest (location 0°11'22"S, 78°31'17.2"W DMS); *D.urcu* (QCAZ-I 1755), Cruz Loma Forest (location 0°11'22"S, 78°31'17.2"W DMS) and *D.valenteae* (QCAZ-I 3142), Sangolquí Canton (location 0°19'59.3"S, 78°25'51"W DMS).

All species were provided by the Evolutionary Genetics Laboratory of Pontificia Universidad Católica del Ecuador. The flies were maintained in banana culture medium supplemented with fresh fruit, in a temperate room at 17 °C, with a 12 h light/dark cycle.

### ﻿Chromosome plates

The metaphase nuclei of cerebral ganglia were obtained from third-instar larvae (ten males, ten females) of each species. Chromosomal plates were prepared by the cell suspension method (Cardoso and Dutra 1979) and thermic shock (Holmquist 1975) and stained with Giemsa. Ten metaphase nuclei were observed for each sex and species. A Ziess Axioskop 2 plus – HAL 100 microscope and a Cannon PowerShot A640 camera (100× objectives lens and optovar 2×) were used to observe and take the pictures of the mitotic chromosome cells. The modal number was considered the chromosome number of each species.

### ﻿Mitotic chromosome analysis

For each species, the total length (TL), relative length (RL) and centromeric index (CI) of the chromosomes were estimated using the Axio Vision 4.4. Standard deviation of relative length was analysed using the SPSS statistical package 26.0v (Table 1).

**Table 1.** Measurement of metaphase chromosomes of five Andean *Drosophila* species.

Species	Chromosome	TL (μm)	RL (%)	CI	SD (n = 10)	Morphology
*D.ecuatoriana*	X	2,49	24,22	0,47	0,27	metacentric
2n = 10	Y	1,85	17,99	0,05	0,03	telocentric
	2	1,65	16,05	0,49	0,12	metacentric
	3	1,54	14,98	0,06	0,19	telocentric
	4	1,42	13,81	0,07	0,21	telocentric
	5	1,33	12,93	0,08	0,16	telocentric
*D.valenteae*	X	2,09	27,42	0,37	0,23	submetacentric
2n = 8	Y	1,73	22,7	0,06	0,31	telocentric
	2	1,4	18,37	0,07	0,21	telocentric
	3	1,26	16,53	0,08	0,23	telocentric
	4	1,14	14,96	0,09	0,14	telocentric
*D.cashapamba*	X	2,88	26,2	0,38	0,12	submetacentric
2n = 6	Y	1,94	17,65	0,05	0,04	telocentric
	2	3,21	29,2	0,47	0,11	metacentric
	3	2,96	26,93	0,49	0,12	metacentric
*D.ninarumi*	X	1,71	27,49	0,46	0,25	metacentric
2n = 10	Y	1,59	25,56	0,06	0,04	telocentric
	2	1,12	18	0,09	0,26	telocentric
	3	0,95	15,27	0,11	0,18	telocentric
	4	0,83	13,34	0,12	0,2	telocentric
	5	0,02	0,32	0,05	0,01	dot
*D.urcu*	X	3,09	24,75	0,48	0,23	metacentric
2n = 12	Y	2,65	21,23	0,04	0,07	telocentric
	2	1,62	12,98	0,49	0,17	metacentric
	3	1,58	12,66	0,06	0,27	telocentric
	4	1,45	11,61	0,07	0,21	telocentric
	5	1,21	9,69	0,08	0,14	telocentric
	6	0,88	7,05	0,11	0,29	telocentric

TL: Total Length, RL: Relative Length, CI: Centromeric Index, SD: Standard deviation.

## ﻿Results

### ﻿The description of new karyotypes of *Drosophila* species is presented below:

**The *Drosophilaguarani* group**

**Figure 1.**
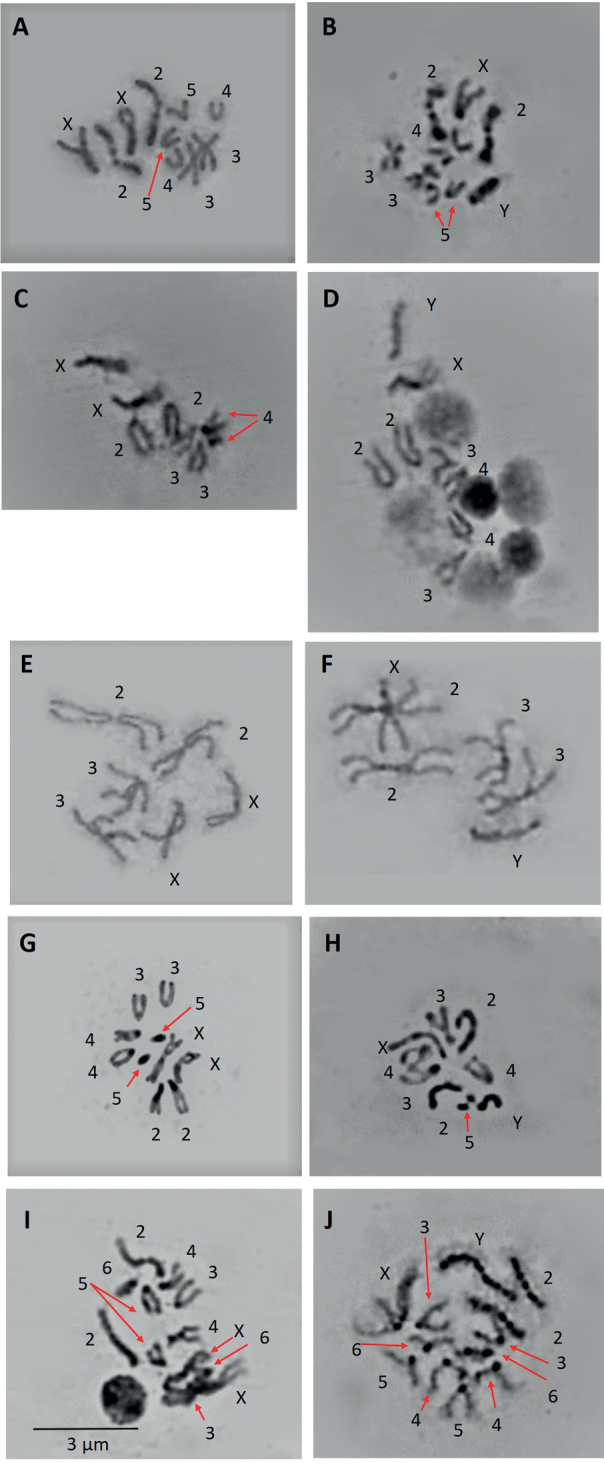
Metaphase karyotype of **A***D.ecuatoriana* female **B***D.ecuatoriana* male **C***D.valenteae* female **D***D.valenteae* male **E***D.cashapamba* female **F***D.cashapamba* male **G***D.ninarumi* female **H***D.ninarumi* male **I***D.urcu* female **J***D.urcu* male. Scale bar: 3 µm (**A–J**).

The karyotype of *D.ecuatoriana* is 2n = 10 (3R, 2V), comprising of four autosomes – a large V-shaped metacentric (pair 2) and three pairs of rod-shaped telocentric chromosomes (pairs 3, 4 and 5) – and the sexual pair (X = V, Y = R). The X chromosome is V-shaped metacentric and the Y chromosome is rod-shaped telocentric (Fig. 1A, B, Table 1).

The karyotype of *D.valenteae* is 2n = 8 (3R, 1J), comprising of three rod-shaped telocentric autosomes (pairs 2, 3 and 4), and the sexual pair (X = J, Y = R). The X chromosome is J-shaped submetacentric, and the Y chromosome is rod-shaped telocentric (Fig. 1C, D, Table 1).

#### ﻿The *Drosophilamesophragmatica* group

The karyotype of *D.cashapamba* is 2n = 6 (2V, 1J) comprising of two V-shaped metacentric autosomes (pairs 2 and 3) and the sexual pair (X = J, Y = R). The X chromosome is J-shaped submetacentric and the Y chromosome is rod-shaped telocentric (Fig. 1E, F, Table 1).

#### ﻿The *Drosophilatripunctata* group

The karyotype of *D.ninarumi* is 2n = 10 (3R, 1V, 1D), comprising of four autosomes – three rod-shaped telocentric (pairs 2, 3 and 4) and one pair of dot-shaped chromosomes (pair 5), and the sexual pair (X = V, Y = R). The X chromosome is V-shaped metacentric and the Y chromosome is rod-shaped telocentric (Fig. 1G, H, Table 1).

The karyotype of *D.urcu* is 2n = 12 (4R, 2V) comprising of five autosomes – a pair of V-shaped metacentric (pair 2) and four pairs of rod-shaped telocentric chromosomes (pairs 3, 4, 5 and 6) – and the sexual pair (X = V, Y = R). The X chromosome is V-shaped metacentric and the Y chromosome is rod-shaped telocentric (Fig. 1I, J, Table 1).

## ﻿Discussion

Considering the high diversity of *Drosophila* species in the Neotropical region little is known about diploid chromosome numbers of these species.

In the *Drosophilaguarani* group, the most common karyotype is 2n = 12. In the present study, the karyotype of *D.ecuatoriana* is 2n = 10 (Fig. 1A, B). A similar 2n = 10 karyotype was reported in other species of this group: *D.guaraja* King, 1947 (King 1947), *D.butantan* (Ratcov et al. 2017) and *D.sachapuyu* (Vela and Villavicencio 2021). The karyotype of *D.valenteae* is 2n = 8 (Fig. 1C, D) and is similar to *D.alexandrei* Cordeiro, 1951 (Cordeiro 1951), both species present the lowest diploid chromosome reported for the *Drosophilaguarani* species group.

Several reports have shown that the karyotype of *Drosophila* species of the *D.mesophragmatica* group is highly conserved, 2n = 10, including a pair of rod-shaped or a dot-like fifth chromosomes (Brncic 1957). Additionally, paracentric inversions are the principal chromosomal rearrangements attributed to this species group (Brncic and Koref 1957). In our study, the chromosome number of *D.cashapamba* is 2n = 6, the chromosomes are large and present a small pericentromeric heterochromatin (Fig. 1E, F). It has been suggested that *D.cashapamba* is a junior synonym of *D.dreyfusi* Dobzhansky et Pavan, 1943 (Dr Carlos Vilela, pers. communication) due to the similarity of the male genitalia and the same chromosome number, 2n = 6 (Dobzhansky and Pavan 1943). However, in this study we maintain the current taxonomical classification until new taxonomic studies confirm the junior synonym status of *D.cashapamba*.

According to the information available in the *Drosophila* karyotype databases (Morelli et al. 2022), the chromosome number 2n = 6 is rarely reported in Drosophila subgenus. Only thirteen species of Drosophila subgenus present three pairs of chromosomes: *D.canalinea* Patterson et Mainland, 1944 from *D.canalinea* group, *D.dreyfusi* and *D.wingei* Cordeiro, 1964 from *D.dreyfusi* group, *D.albomicans* Duda, 1923, *D.annulipes* Duda, 1924, *D.neohypocausta* Lin et Wheeler, 1973 from *D.immigrans* group, *D.atalaia* Vilela et Sene, 1982 from *D.peruensis* group, *D.pinicola* Sturtevant, 1942 from *D.pinicola* group, *D.quinaria* Loew, 1866 from *D.quinaria* group; *D.neoguaramunu* Frydenberg, 1956 from *D.tripunctata* group, *D.montana* Patterson et Wheeler, 1942 from *D.virilis* group, *D.aracea* Heed et Wheeler, 1957 and *D.tranquilla* Spencer, 1942 (not grouped).

Most species of the *D.tripunctata* group have a karyotype 2n = 12, the sixth pair is a dot chromosome; some members of *D.tripunctata* group have a karyotype 2n = 10 (Morelli et al. 2022). In the karyotype of *D.ninarumi*, 2n = 10, it is present a dot-like fifth pair of chromosome (Fig. 1G, H) which is reported in the most species of *Drosophilatripunctata* group. This karyotype is similar to *D.fairchaldi* Pipkin et Heed, 1964 and *D.unipunctata* Patterson, 1943 (Wharton 1943; Pipkin and Heed 1964; Clayton and Wheeler 1975) but in these species the dot-like chromosome is absent. In the case of *D.urcu*, the karyotype is 2n = 12, all the chromosomes are large metacentric or telocentric (Fig. 1I, J). Our data show that the karyotype of *D.ninarumi* and *D.urcu* have a relevant similitud, the sexual chromosomes are the largest of the chromosome set, with a Y chromosome heteropycnotic (Fig. 1G, J).

Traditional studies like genetic crosses, in situ hybridization, polytene chromosomes maps or karyotype description are not commonly performed. However, for the genus *Drosophila*, the information provided by cytological studies is the initial tool in understanding the evolutionary history and the high radiation of the *Drosophila* species in the Neotropical region and also important in the beginning of genomic studies on these species.

## ﻿Conclusions

This study reveals the first karyotype description of five Neotropical species of *Drosophila*. Only the karyotype of *D.urcu*, 2n = 12, is similar to the ancestral karyotype of *Drosophila*, but the sixth pair are large chromosomes. The karyotypes of *D.ecuatoriana* and *D.ninarumi* are 2n = 10, but only the last one has a dot-like chromosome. The karyotype of *D.valenteae* is 2n = 8; this is the second species of *D.guarani* group that have this chromosome number. The karyotype of *D.cashapamba* presents a low chromosome number, 2n = 6, which is only reported in other thirteen species of subgenus Drosophila.
